# Attitude of trainees toward tribal entrepreneurship training on solar lantern development in Ladakh: a descriptive study

**DOI:** 10.3389/fsoc.2026.1802017

**Published:** 2026-05-15

**Authors:** Mohd Rafee, Surendra Kumar Gotherwal, Tsewang Dolma, Anamika Sharma, Ruhana Rafiq, Stanzin Khenrab

**Affiliations:** 1 Department of Commerce and Management, University of Ladakh, Leh, India; 2 Ministry of Electronics and Information Technology (MeitY), New Delhi, India; 3 G.B. Pant University of Agriculture and Technology, Pantnagar, India; 4 Sher-e-Kashmir University of Agricultural Sciences and Technology of Jammu, Jammu, India; 5 Krishi Vigyan Kendra-Leh, University of Ladakh, Leh, India

**Keywords:** employment generation, Ladakh, renewable energy, skill development, tribal entrepreneurship, vocational training

## Abstract

**Introduction:**

While renewable energy skill development programmes have expanded across India's peripheral regions, limited empirical evidence exists on how tribal trainees in geographically isolated settings perceive such initiatives and the factors that shape their entrepreneurial attitudes. This study examines the attitudes of 320 Scheduled Tribe trainees towards a Solar Lantern Development entrepreneurship training programme in Ladakh.

**Methods:**

Drawing on the Theory of Planned Behavior, the Competence-Confidence framework and institutional perspectives on peripheral entrepreneurship, the study employs a cross-sectional descriptive research design. Attitudes were measured using a pilot-tested 20-item Likert scale (Cronbach's Alpha = 0.852) and a post-training technical knowledge assessment. Descriptive statistics, independent samples t-tests, Pearson correlation and multiple linear regression were used for analysis.

**Results:**

The findings indicate an overall positive attitude with the knowledge score demonstrating a strong positive association with entrepreneurial attitude (*r* = 0.831, *p* < 0.01). Multiple linear regression confirmed technical knowledge (β = 0.969, *p* < 0.001) as the strongest predictor of attitude (R^2^ = 0.765) while age showed a significant negative association (β = −0.161, *p* < 0.001). However, logistical constraints, limited market linkages and household responsibilities emerged as key barriers identified through item-level mean percent score analysis.

**Discussion:**

The findings are interpreted as associations rather than causal effects, given the cross-sectional design and purposive sampling. The study contributes to the literature on green skills development in peripheral regions by demonstrating that technical competence is a necessary but insufficient condition for entrepreneurial motivation and that institutional support structures are critical for translating skills into enterprise formation.

## 1 Introduction

Globally, energy policy discourse has increasingly shifted toward decentralized renewable solutions, particularly in regions where extending central electricity grids is technically challenging or economically unviable. Ladakh, a high-altitude Himalayan region characterized by rugged terrain, harsh climatic conditions, and dispersed settlements exemplifies such challenges. Conventional grid-based electricity remains unreliable or inaccessible in several remote villages (Gergan, 2013). Simultaneously, the region benefits from more than 300 days of sunshine annually, making solar energy a highly suitable alternative (Ladakh Renewable Energy Development Agency, 2011; Ministry of New and Renewable Energy, 2022). India's broader transition toward decentralised renewable energy provision underscores the increasing relevance of community-level solar initiatives (Hiremath et al., 2013; Tripathi et al., 2016).

Recognizing this potential, policy approaches in India have gradually evolved from the mere distribution of solar products toward fostering locally embedded renewable energy entrepreneurship. Skill development initiatives now aim to train local youth in assembling, servicing and marketing solar technologies thereby addressing energy access deficits while creating sustainable livelihood opportunities (International Renewable Energy Agency, 2022; Hande and Williams, 2005). This shift aligns with the broader global emphasis on “green skilling” within vocational education and training (VET) frameworks which emphasize the integration of environmental sustainability with employment generation (Pavlova, 2018; McGrath and Powell, 2016).

Understanding the effectiveness of such programs requires a multi-layered theoretical perspective. At the individual level, the Theory of Planned Behavior (TPB) posits that attitudes, subjective norms, and perceived behavioral control jointly determine behavioral intentions, including the intention to initiate entrepreneurial ventures (Ajzen, 1991). This framework has been widely applied in studies of technology adoption and entrepreneurial intention (Smith, 2019; Annu et al., 2021). However, TPB alone is insufficient for explaining entrepreneurship in peripheral and resource-constrained contexts where structural barriers-such as market access, institutional support and infrastructure-often override individual-level attitudinal factors (Tikly, 2013; Latchem, 2017). Accordingly, this study complements TPB with the Competence-Confidence framework (Genc and Akilli, 2019; Bandura, 2012) which posits that technical mastery is a prerequisite for entrepreneurial self-efficacy and with institutional perspectives that emphasize the role of post-training support ecosystems, market linkages and community-level institutions in translating skills into enterprise outcomes (Lackéus, 2015; Sharma and Singh, 2023). This integrated approach allows the study to examine not only individual attitudes but also the structural and institutional factors that facilitate or constrain entrepreneurial behavior in tribal settings.

Despite the strategic importance of solar entrepreneurship training in remote tribal regions, there is a notable gap in the empirical literature. Existing studies on renewable energy training attitudes have predominantly focused on non-tribal, non-peripheral populations (Kumar and Godara, 2017; Yadav et al., 2009) and few have examined the interplay between individual competence, institutional support and entrepreneurial attitude formation among Scheduled Tribe communities in geographically isolated settings. Addressing this gap, the present study analyses trainee profiles, attitudes, perceived challenges and the association between technical knowledge and entrepreneurial readiness among 320 Scheduled Tribe trainees in Ladakh. By integrating individual-level attitudinal analysis with an examination of structural barriers, the study contributes to the literature on green skills development in peripheral regions, offering insights into the conditions under which decentralized skill development initiatives can support inclusive and sustainable livelihoods.

## 2 Methods

### 2.1 Research design and sampling

A cross-sectional descriptive research design was employed which is commonly used for studies examining attitudes and perceptions in a defined population at a single point in time (Etikan et al., 2016). This design is appropriate for establishing the distribution of attitudes and identifying associated factors however, it does not support causal inference. Accordingly, all relationships reported in this study are interpreted as associations rather than causal effects.

The study was conducted in the Union Territory of Ladakh. The target population comprised Scheduled Tribe trainees who had participated in solar lantern development training programs. Using purposive sampling, 320 respondents were selected based on the criterion that they had completed the full duration of the training module ensuring adequate exposure to program content. Purposive sampling was chosen because the study required access to a specific, hard-to-reach population (tribal trainees in remote Ladakh) for whom probability-based sampling was logistically infeasible. This sampling approach may limit the generalizability of findings to the broader population of potential trainees and introduces a potential selection bias as only those who completed the training were included. Trainees who dropped out may have held systematically different attitudes and this should be considered when interpreting the results.

### 2.2 Research instrument

Data were collected using a structured interview schedule consisting of three sections. The first section captured socio-demographic characteristics (age, gender, education level, and occupation). The second section comprised a 20-item attitude scale developed based on a review of existing literature on vocational training attitudes and renewable energy adoption (Smith, 2019; Annu et al., 2021; Kumar and Godara, 2017). The scale included 15 positive statements related to training quality and entrepreneurial outcomes (e.g., “The training has increased my confidence to start a solar lantern business,” “The trainers explained the concepts clearly”) and five negative statements addressing logistical and technical barriers (e.g., “I had difficulty understanding technical terms used during the training,” “Household responsibilities affected my participation”). Responses were recorded on a five-point Likert scale ranging from 1 (strongly disagree) to 5 (strongly agree) with negative items reverse-scored, in accordance with established conventions in attitude measurement research (Eagly and Chaiken, 1993). The total attitude score was computed by summing individual item scores and converting to a percentage scale (range: 20–100). Content validity was established through expert review by three subject matter specialists (two from MeitY and one from the Department of Commerce, University of Ladakh) who evaluated the relevance, clarity and cultural appropriateness of each item. The instrument was subsequently pilot-tested with 30 trainees drawn from a non-overlapping cohort, demonstrating high internal consistency (Cronbach's Alpha = 0.852, Standardized Alpha = 0.869, and Guttman Lambda 6 = 0.915).

The third section consisted of a post-training technical knowledge assessment comprising 20 multiple-choice questions covering solar cell principles, lantern assembly procedures, battery maintenance and troubleshooting. The assessment was developed in consultation with subject matter experts from MeitY and pilot-tested for clarity and difficulty level with the same pilot cohort. Item difficulty indices ranged from 0.35 to 0.82, confirming adequate discrimination. Scores were expressed as a percentage (0–100). This combined approach aligns with competence-confidence frameworks that emphasize the role of technical mastery in shaping self-efficacy and entrepreneurial motivation (Bandura, 2012; Genc and Akilli, 2019).

As both the attitude and knowledge measures were collected from the same respondents at the same time point using self-report instruments common method bias (CMB) is a potential concern. To mitigate this, several procedural remedies were implemented: (a) the attitude and knowledge sections were clearly separated in the instrument with the knowledge section comprising objective multiple-choice questions rather than self-assessments; (b) anonymity was assured to reduce social desirability bias and (c) negatively worded items were included to reduce acquiescence bias. While these measures reduce the likelihood of CMB the possibility cannot be entirely eliminated in a single-source, cross-sectional design and this is acknowledged as a limitation.

### 2.3 Data analysis

Data were analyzed using R statistical software (R Core Team, 2021). Reliability was assessed using Cronbach's Alpha, Standardized Alpha, and Guttman Lambda 6. Descriptive statistics (mean, standard deviation) were used to summarize trainee characteristics and attitude scores with skewness and kurtosis computed to assess distributional properties and justify the use of parametric tests. Independent samples *t*-tests were conducted to examine gender differences. Pearson correlation coefficients were computed to assess bivariate associations between predictor variables and attitude scores. Multiple linear regression analysis was employed to identify the relative contribution of age, education level and knowledge score as predictors of entrepreneurial attitude with the coefficient of determination (*R*^2^) reported to quantify explained variance. Barriers were identified through item-level mean percent score (MPS) analysis wherein individual statement means were ranked to distinguish program strengths from weaknesses. It is important to note that the regression coefficients reported are unstandardized (β) and the term “predictor” is used in the statistical sense and does not imply causation.

### 2.4 Ethical considerations

The study involved a non-invasive evaluation of a government-supported training program and did not collect sensitive personal data. Participation was voluntary and informed verbal consent was obtained from all participants prior to data collection. Participants were informed of the study objectives and their right to withdraw at any stage without any negative consequences. The study complied with institutional and national research ethics guidelines and formal ethical approval was not required in accordance with institutional policy.

## 3 Results and discussion

### 3.1 Socio-demographic composition

The demographic characteristics of the sample provide key insights into the reach and limitations of the training program. The program primarily attracted younger participants: 22.50% fell within the 15–20 years age group while 63.44% were in the 21–30 years age group (Table 1). The involvement of youth suggests that the program is successfully positioning solar entrepreneurship as a feasible career option for new entrants to the workforce.

**Table 1:** Socio-demographic profile of respondents (*n* = 320).

Variable	Category	Frequency (*f*)	Percentage (%)
Age group	15–20	72	22.50
	21–30	203	63.44
	31–40	42	13.12
	41–50	3	0.94
Gender	Male	233	72.81
	Female	87	27.19
Education	Secondary	42	13.12
	Higher secondary	113	35.31
	Graduation	138	43.13
	Post-graduation and above	27	8.44
Occupation	Student	260	81.25
	Working/other	60	18.75

A notable feature of the sample is that 81.25% were students, indicating that the program functions primarily as a supplementary skill-building course alongside formal education. This student-dominated composition has important implications for interpreting the findings. Students may exhibit higher baseline enthusiasm for learning and more favorable attitudes toward training as they are already in an educational mindset compared to working adults or unemployed youth for whom the stakes of entrepreneurial success may be higher. Additionally, students may face fewer immediate economic pressures potentially inflating attitude scores relative to what might be observed in a more diverse occupational sample. These compositional characteristics should be considered when generalizing the findings as the attitudes of students undergoing supplementary training may not fully represent those of the broader target population for renewable energy entrepreneurship programs.

Educational qualifications were varied with 43.13% holding graduation degrees followed by 35.31% with higher secondary education (Table 1). The gender distribution showed a notable disparity with males comprising 72.81% of participants compared to 27.19% females. This pattern reflects broader structural inequalities in access to technical training in rural India (Baruah, 2015) and suggests a need for targeted engagement strategies. The predominance of youth (85.94% aged below 30 years) aligns with India's demographic dividend framework for youth-centric skilling (Ministry of Human Resource Development, 2023).

### 3.2 Reliability and descriptive statistics

Reliability analysis confirmed high internal consistency of the attitude scale. Cronbach's Alpha was 0.852, exceeding the standard acceptable threshold of 0.70 (Tavakol and Dennick, 2011). The standardized alpha (0.869) and Guttman Lambda 6 (0.915) further corroborated this finding (Table 2).

**Table 2:** Reliability coefficients of the attitude scale.

Metric	Value	Interpretation
Cronbach's alpha	0.852	High reliability
Standardized alpha	0.869	High reliability
Guttman lambda 6	0.915	High reliability

The mean total attitude score was 79.78 (SD = 7.37) out of a maximum possible score of 100, with a median of 79.00 (Table 3). The score distribution exhibited slight negative skewness (−0.18) and a kurtosis value of 3.04 indicating an approximately normal (mesokurtic) distribution. This near-normal distribution supports the use of parametric statistical tests in subsequent analyses.

**Table 3:** Descriptive statistics of total attitude scores (*n* = 320).

Statistic	Value
Mean ± SD	79.78 ± 7.37
Median	79.00
Minimum	61.00
Maximum	97.00

### 3.3 Attitude classification

Respondents were classified into three attitude categories using the Mean ± SD approach: scores below (Mean–SD) were classified as “Unfavorable,” scores within (Mean ± SD) as “Neutral” and scores above (Mean + SD) as “Favourable” (Table 4). This statistical classification while standard in attitudinal research warrants critical interpretation. The “neutral” category represents the statistical middle of this specific sample's distribution not an absolute benchmark of moderate satisfaction. Given the high mean score (79.78/100), respondents classified as “neutral” in fact held generally positive attitudes, this label reflects their position relative to peers rather than an absence of opinion.

**Table 4:** Distribution of respondents by attitude category (*n* = 320).

Attitude category	Frequency (*f*)	Percentage (%)
Unfavorable (< 72.41)	34	10.62
Neutral (72.41–87.15)	228	71.26
Favorable (>87.15)	58	18.12

**Figure 1:**
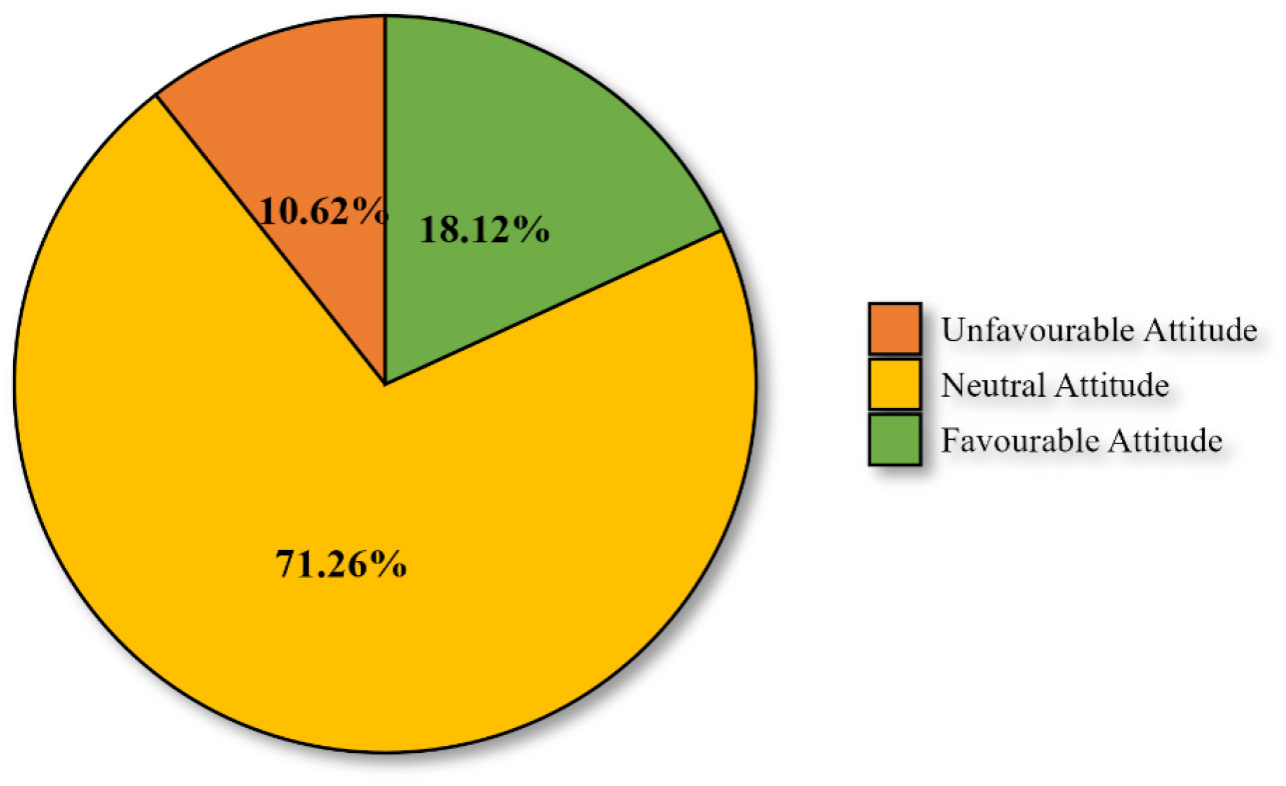
Distribution of respondents by attitude category.

The distribution of respondents across favorable, neutral and unfavorable attitude categories is presented in Figure 1.

Moreover, from a local cultural perspective the large neutral group (71.26%) may reflect a “wait-and-see” disposition rather than active satisfaction. In indigenous communities a neutral response may signal cautious skepticism regarding market viability and post-training support rather than contentment with the program itself. This aspect warrants qualitative exploration in future research. The low percentage of unfavorable respondents (10.62%) indicates very low active dissatisfaction. The low item-level score on networking (MPS = 44.00) highlights a gap in the post-training ecosystem: trainees acquired assembly competencies but felt unsupported regarding market linkages, confirming (Lackéus 2015) argument that entrepreneurial education is incomplete without venture creation skills.

### 3.4 Item-wise analysis: strengths and barriers

**Figure 2:**
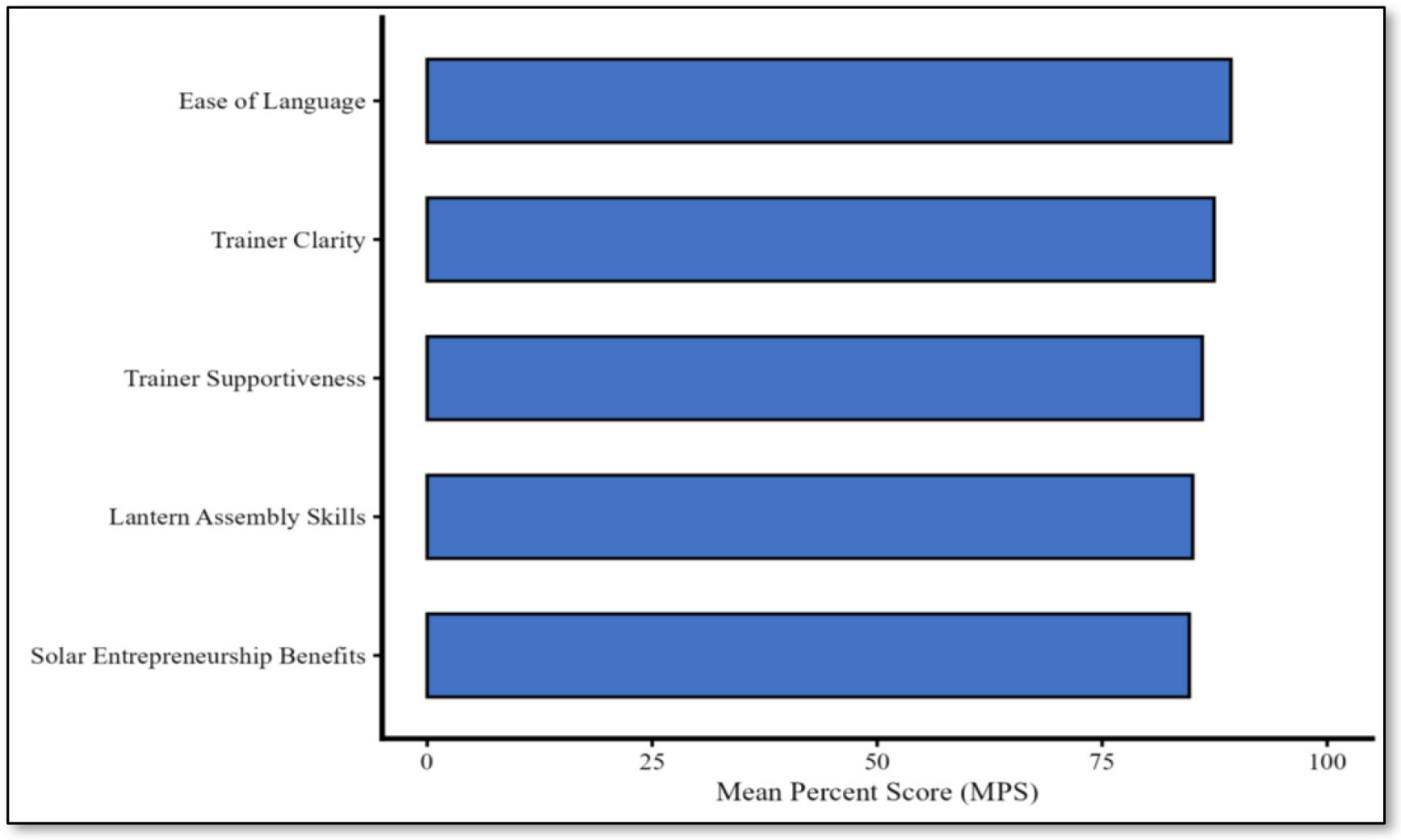
Top 5 highest ranked training aspects.

A detailed item-level analysis identifies specific program strengths and weaknesses. The primary drivers of positive attitude were related to pedagogical quality: ease of language ranked first (MPS = 89.31) and trainer clarity ranked second (MPS = 87.44; Table 5; Figure 2). This finding is significant for a tribal development program as it demonstrates effective adaptation of instructional approaches to local linguistic and cultural contexts. The trainers successfully moved beyond complex technical jargon presenting material accessibly. This success aligns with critical pedagogy principles regarding the reduction of linguistic barriers in technical training (Skutnabb-Kangas, 2000) and resonates with (Latchem 2017) emphasis on contextualized pedagogical approaches in VET for marginalized communities. Such findings also reinforce the broader argument that vocational qualifications must align pedagogy with both employment and further-education pathways (Wheelahan and Moodie, 2017).

**Table 5:** Evaluation of training efficacy: a ranked assessment (*n* = 320).

Statement	Mean score (1–5)	MPS (%)	Rank
Ease of language	4.47	89.31	1
Trainer clarity	4.37	87.44	2
Trainer supportiveness	4.31	86.12	3
Lantern assembly skills	4.25	85.06	4
Solar entrepreneurship benefits	4.23	84.69	5
Renewable energy awareness	4.22	84.38	6
Motivation level	4.21	84.25	7
Availability of materials	4.16	83.19	8
Knowledge of government schemes	4.15	82.94	9
Venue comfort and equipment	4.14	82.81	10
Inclusive environment	4.13	82.69	11
Confidence to train others	4.13	82.62	12
Guidance on earning	4.07	81.38	13
Appropriateness of duration	3.99	79.88	14
Business start-up confidence	3.97	79.44	15
Difficulty with technical terms	2.82	56.31	16
Weather disruptions	2.42	48.31	17
Household responsibilities impact	2.25	45.06	18
Access to networks	2.20	44.00	19
Transport issues	1.75	34.94	20

The analysis also highlights structural barriers. Transport issues ranked lowest (MPS = 34.94), reflecting Ladakh's challenging geography. However, this low score on a negative statement is a positive finding: transportation was effectively mitigated by pick-up and drop-off services coordinated through local village structures. Access to networks ranked 19th (MPS = 44.00) confirming a critical gap in the post-training ecosystem. This finding resonates with the “Last Mile” challenges emphasized by the (International Energy Agency 2023) and underscores that from an institutional perspective, the program succeeded in skill transfer but fell short in facilitating the market linkages and institutional support necessary for enterprise formation (Sharma and Singh, 2023).

**Figure 3:**
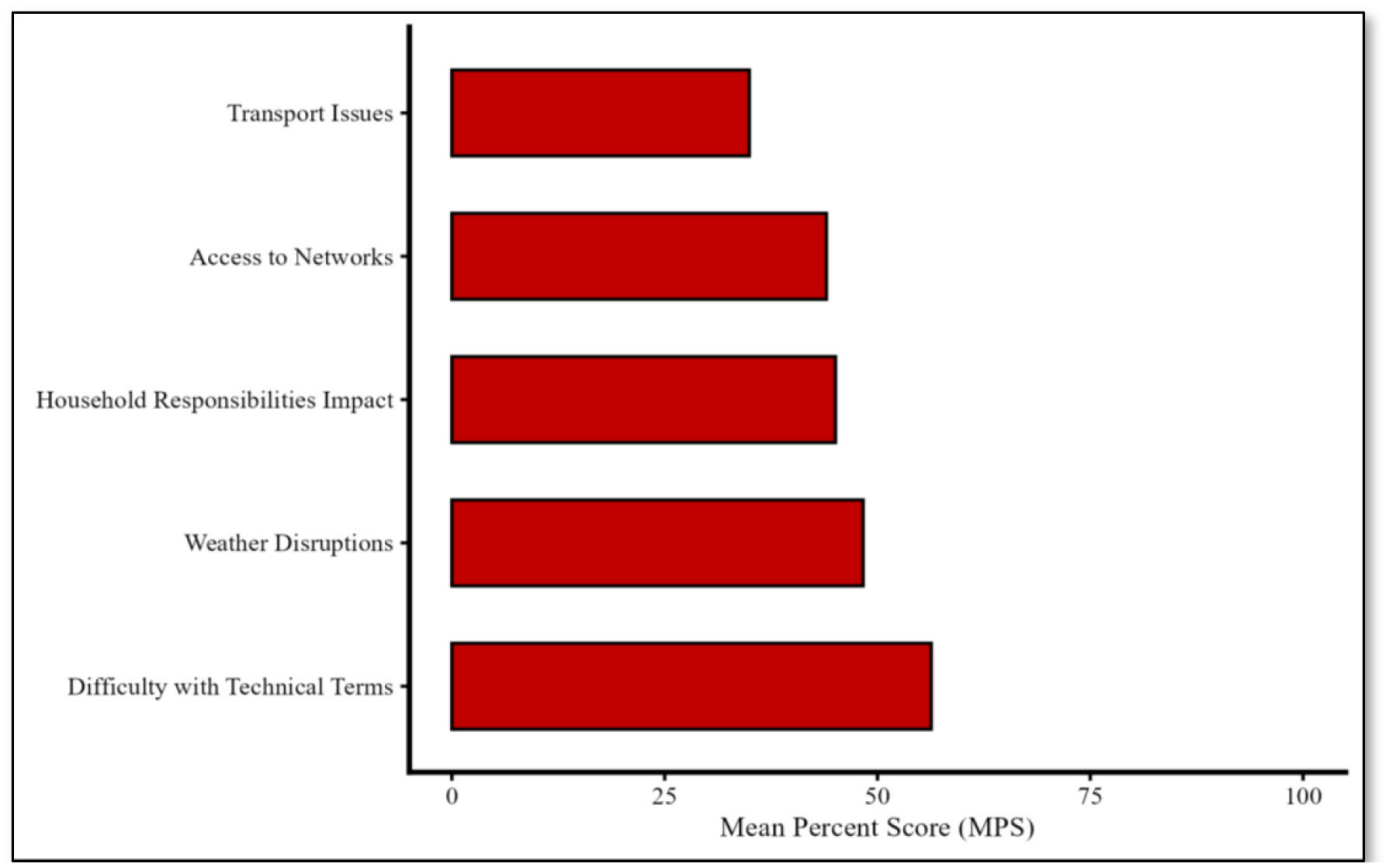
Top 5 lowest ranked training aspects (barriers).

Figure 3 further analyses the associated barriers by severity. The findings indicate that, although technical difficulty posed a moderate challenge, logistical issues such as weather and transport emerged as the most significant constraints.

**Figure 4:**
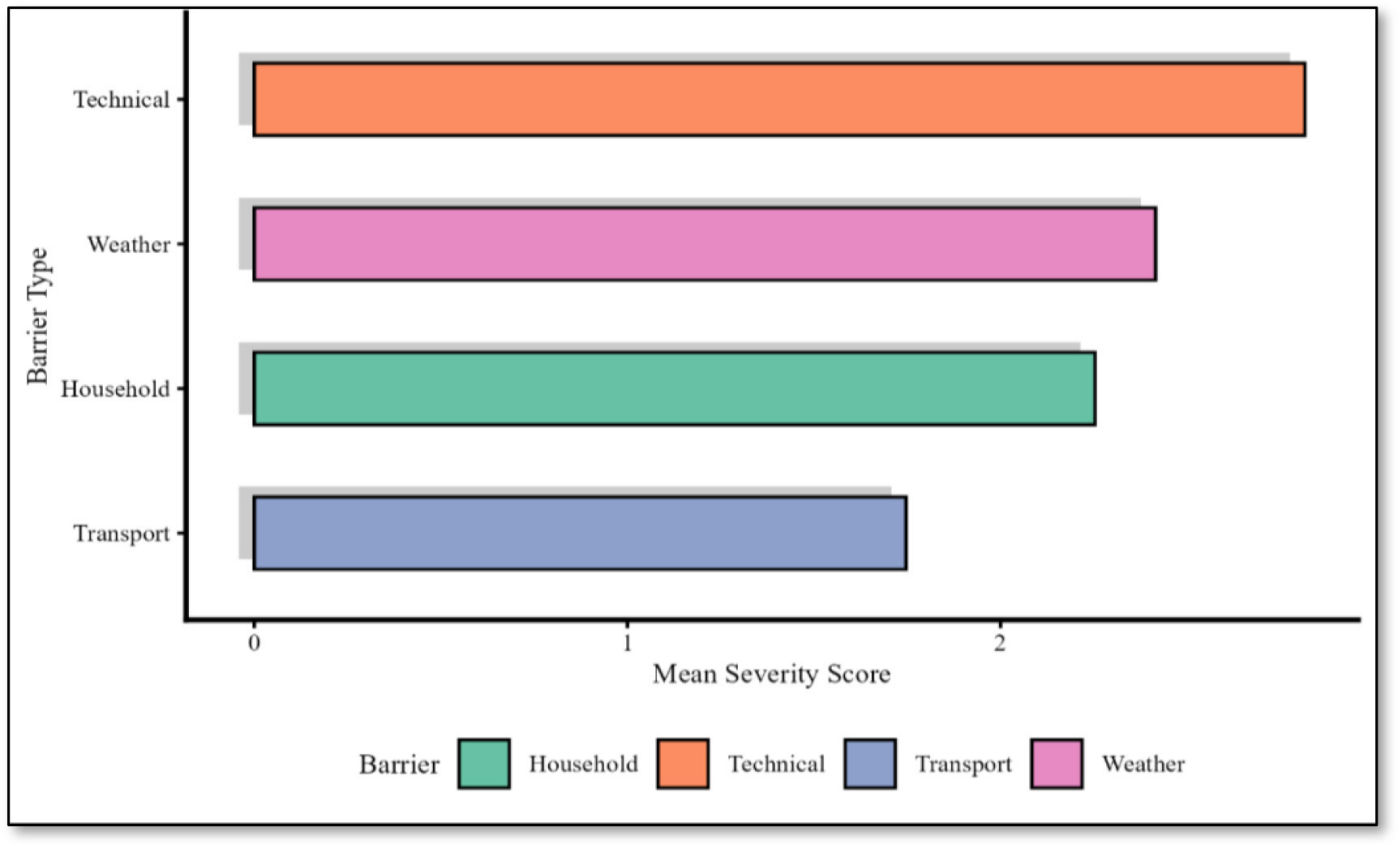
Severity of barriers faced (1–5 Scale).

The severity of barriers faced by the trainees on a five-point scale is shown in Figure 4.

### 3.5 Gender comparison

**Figure 5:**
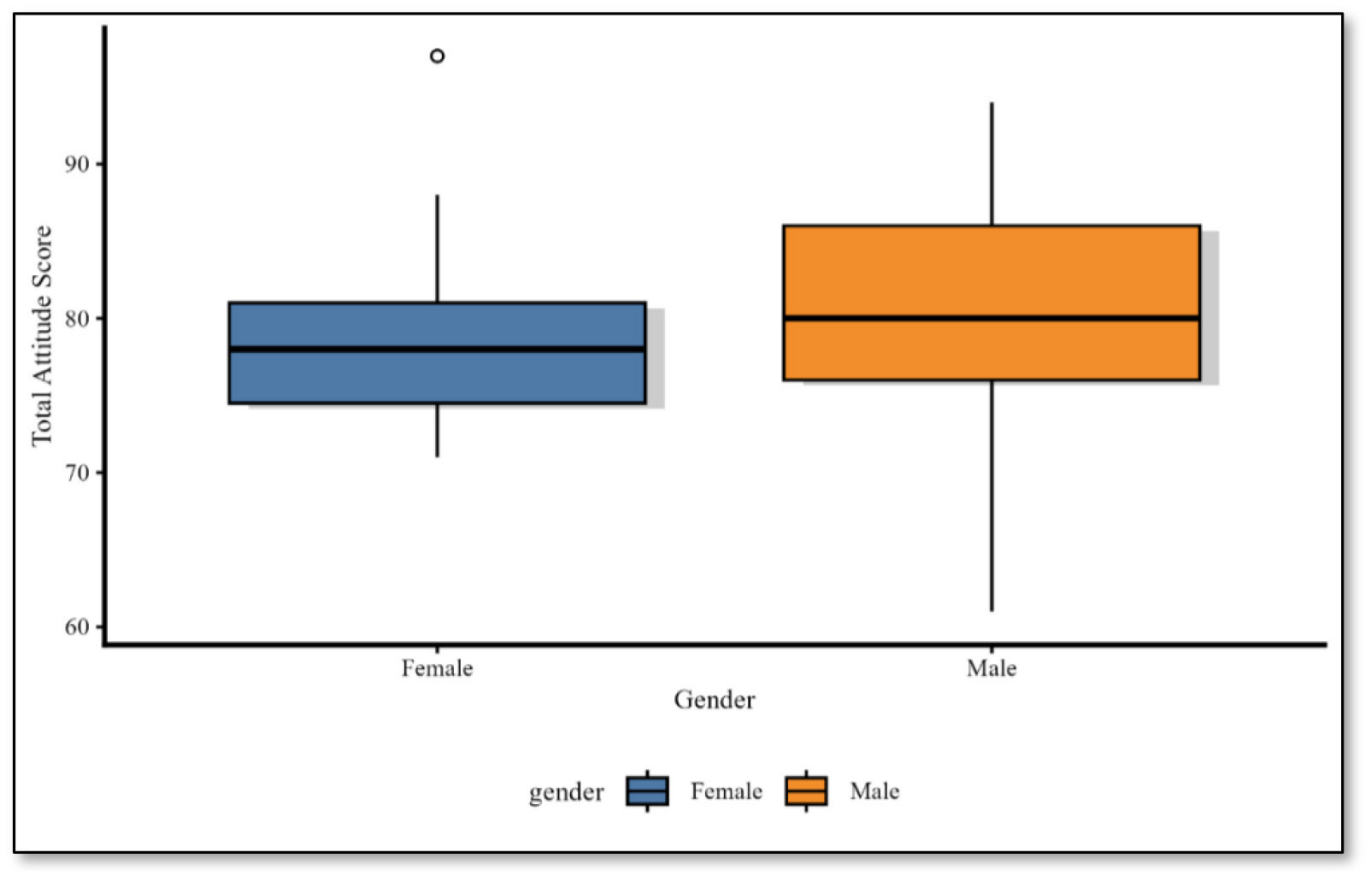
Attitude score distribution by gender. The figure presents box plots for Female vs. Male scores. The male median is slightly higher and the range is tighter compared to the female distribution.

An independent samples *t*-test examined gender differences in attitude scores. The mean attitude score for males (80.20) was slightly higher than for females (78.64) with the difference approaching statistical significance (*t* = −1.968, *p* = 0.05; Table 6; Figure 5).

**Table 6:** Independent samples *t*-test of attitude scores by gender.

Group	Mean attitude score	*t*-value	*p*-value
Female (*n* = 87)	78.64	−1.968	0.050
Male (*n* = 233)	80.20	−1.968	0.050

This marginally significant difference warrants contextual interpretation. Household responsibilities, ranked 18th as a barrier, suggest that female participants faced greater domestic constraints. This pattern reflects the “double burden” frequently discussed in feminist economics and VET literature: women in rural India are commonly expected to manage domestic responsibilities alongside skill acquisition, resulting in “time poverty” (Baruah, 2015). (Powell and McGrath 2019) argue that without gender-responsive logistical support-such as childcare or flexible timing-standard VET programs inadvertently reinforce gender hierarchies. This structural dimension extends beyond the individual-level attitudinal analysis captured by the present data and underscores the need for institutionally embedded gender-responsive design in future training programs.

### 3.6 Correlation and regression analysis

**Figure 6:**
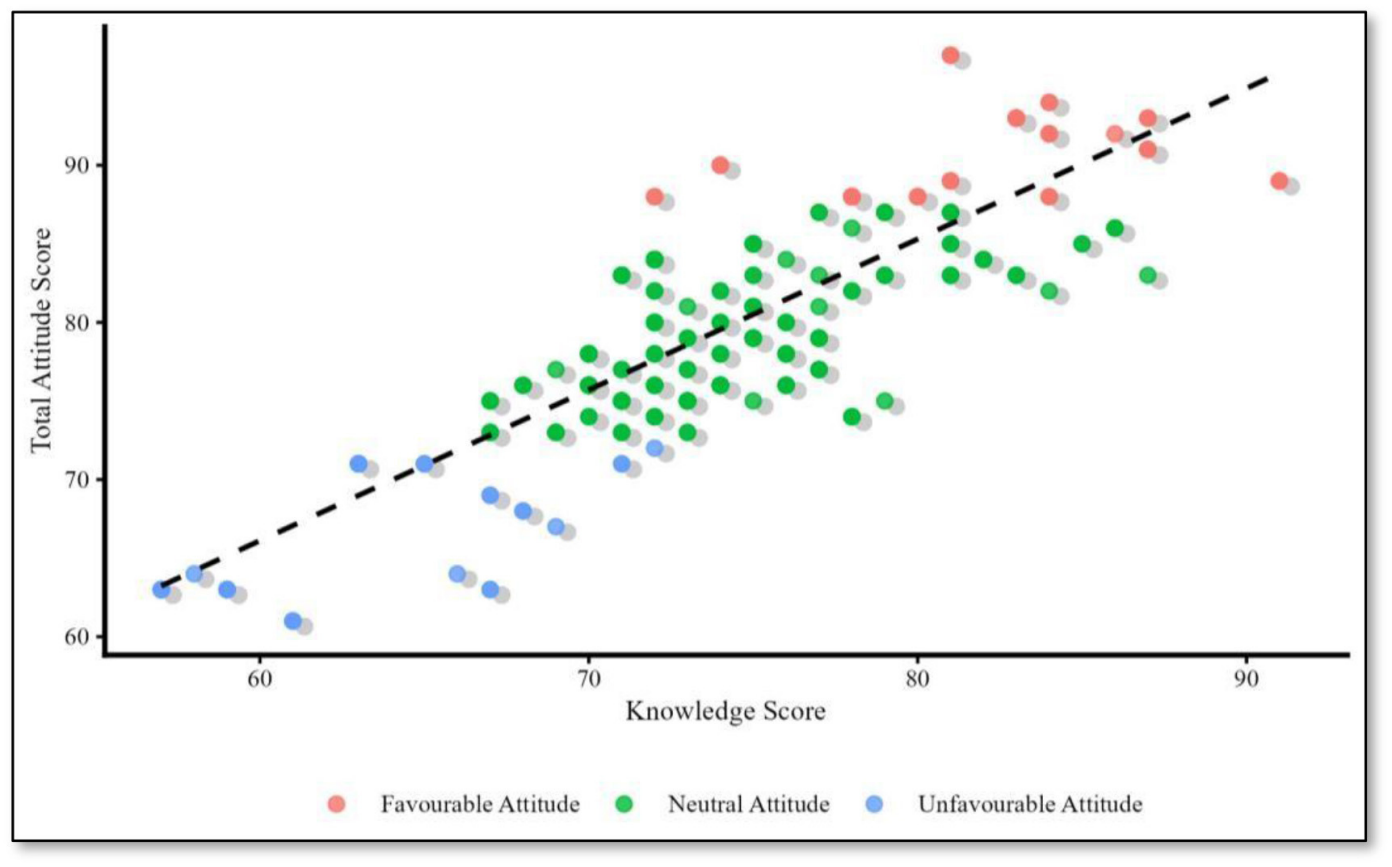
Correlation between knowledge score and attitude.

A Pearson correlation analysis examined bivariate associations between predictor variables and attitude scores. The knowledge score demonstrated a strong, positive and statistically significant association with attitude (*r* = 0.831, *p* < 0.01), while age (*r* = −0.078, *p* > 0.05) and education level (*r* = 0.113, *p* > 0.05) showed negligible and non-significant correlations (Table 7; Figure 6).

**Table 7:** Pearson correlation matrix.

Variable	Correlation coefficient (*r*)	Significance
Age	−0.078	Non-significant (*p* > 0.05)
Education level	0.113	Non-significant (*p* > 0.05)
Knowledge score	0.831	Significant (*p* < 0.01)

Multiple linear regression analysis was conducted to examine the relative contributions of age, education level and knowledge score as statistical predictors of attitude (Table 8). The model was statistically significant (*F* = 342.8, *p* < 0.001) and explained 76.5% of the variance in attitude scores (*R*^2^ = 0.765; Adjusted *R*^2^ = 0.763). The knowledge score emerged as the strongest predictor (β = 0.969, SE = 0.034, *t* = 28.512, and *p* < 0.001) consistent with the Competence-Confidence framework positing that technical mastery is associated with entrepreneurial self-efficacy (Genc and Akilli, 2019; Bandura, 2012). Education level was a significant positive predictor (β = 1.807, SE = 0.314, *t* = 5.761, and *p* < 0.001). Age showed a significant negative association when knowledge was controlled (β = −0.161, SE = 0.041, *t* = −3.957, and *p* < 0.001) suggesting that younger trainees exhibited slightly more favorable attitudes than older trainees with equivalent technical knowledge.

**Table 8:** Multiple linear regression analysis (Dependent variable: attitude score).

Predictor	β-coefficient	Std. error	*t*-value	*p*-value
Age	−0.161	0.041	−3.957	< 0.001
Education level	1.807	0.314	5.761	< 0.001
Knowledge score	0.969	0.034	28.512	< 0.001

*R*^2^ = 0.765, Adjusted *R*^2^ = 0.763, *F* = 342.8, *p* < 0.001.

Coefficients are unstandardized.

“Predictor” is used in the statistical sense and does not imply causation.

It is important to emphasize that these regression results identify statistical associations not causal relationships. The cross-sectional design precludes determination of causal direction for instance, it is plausible that trainees with more favorable pre-existing attitudes were more motivated to acquire technical knowledge rather than knowledge acquisition causing attitudinal change. Furthermore, unmeasured confounders-such as prior exposure to solar technology, household income or social network characteristics-may influence both knowledge and attitude. These limitations are inherent to the research design and are discussed further in Section 3.8.

### 3.7 Synthesis: integrating individual and institutional perspectives

The findings of this study can be situated within a broader framework that integrates individual-level attitudinal factors with structural and institutional dimensions. At the individual level, the strong association between technical knowledge and entrepreneurial attitude is consistent with both the TPB (Ajzen, 1991) in which perceived behavioral control (here operationalized through technical competence) shapes intentions-and the Competence-Confidence framework (Genc and Akilli, 2019; Bandura, 2012) which positions self-efficacy derived from mastery experience as the primary driver of entrepreneurial motivation. The finding that knowledge explains 76.5% of attitude variance underscores that technical competence is a necessary condition for positive entrepreneurial attitudes in this population.

However, item-level analysis reveals that technical competence is not a sufficient condition for entrepreneurial readiness. The low scores on networking (Rank 19) and market access highlight the institutional void that persists after training. This aligns with critiques of purely individual-level frameworks: (Tikly 2013) argues that TVET outcomes in developing countries are fundamentally shaped by structural conditions, including market access, institutional support and the broader political economy. Similarly, (Sharma and Singh 2023) document that the “last mile” challenge in rural renewable energy adoption is not merely a matter of individual capacity but of systemic institutional gaps. In the Ladakhi context, these gaps are magnified by geographic isolation, limited private-sector presence and the absence of formal market intermediaries.

The negative age-attitude association, even after controlling for knowledge adds a further dimension. Older community members may hold traditional knowledge systems that are not adequately acknowledged within a purely technical training curriculum potentially creating a sense of alienation. This suggests that training programs need to move beyond a one-size-fits-all approach and integrate culturally relevant content that bridges traditional and modern knowledge systems.

The gender gap, while only marginally significant in the present data reflects structural inequalities that extend well-beyond individual attitudes. The “double burden” of domestic labor (Baruah, 2015) and the cultural coding of technical spaces as masculine (Powell and McGrath, 2019) represent institutional barriers that cannot be addressed through training design alone but require coordinated policy interventions across education, social protection and local governance domains.

Taken together, these findings suggest that the relationship between training, attitudes and entrepreneurial outcomes is mediated by institutional factors that were measured indirectly in this study through item-level barrier analysis. This is consistent with broader literature on training transfer which emphasises that workplace and contextual conditions are decisive in converting acquired competencies into sustained behavioural change (Burke and Hutchins, 2007). The theoretical contribution of this study lies in demonstrating that in peripheral tribal contexts technical competence is a necessary but insufficient condition for entrepreneurial motivation and that institutional support structures-market linkages, community-based organizations and post-training incubation-are critical for translating competence into enterprise formation. This extends the Competence-Confidence model by embedding it within the structural realities of peripheral entrepreneurship.

### 3.8 Limitations and scope for future research

Several limitations must be acknowledged. First, the cross-sectional design precludes causal inference: all reported relationships are associations. Longitudinal research incorporating pre-training baseline assessments would enable stronger causal claims and would help determine whether positive attitudes translate into actual enterprise formation over time. Second, purposive sampling of completers introduces selection bias: future studies should include non-completers to assess attrition-related attitude differences. Third, the student-dominated sample (81.25%) limits generalizability to broader populations of potential renewable energy entrepreneurs, including unemployed youth and existing informal-sector workers. Fourth, while procedural measures were implemented to mitigate CMB, the single-source, single-time-point design cannot entirely eliminate this concern. Future research should consider using mixed-methods designs incorporating qualitative components (e.g., focus group discussions, semi-structured interviews) to capture the lived experience of trainees-particularly how they perceive solar energy relative to traditional fuel-based energy sources and the cultural dynamics shaping entrepreneurial intentions. Additionally, incorporating objective measures of entrepreneurial outcomes (actual enterprise formation, income generation) and institutional factors (market access indices, community support structures) as separate data sources would strengthen the evidentiary base and address the limitations of self-report data.

## 4 Conclusion and policy implications

This study demonstrates that solar lantern entrepreneurship training is associated with positive attitudes and entrepreneurial confidence among Scheduled Tribe youth in Ladakh. The strong association between technical knowledge and entrepreneurial attitude confirms the relevance of the Competence-Confidence framework in this context while the identification of structural barriers-limited market linkages, absent post-training support and gender-specific constraints-extends the analysis beyond individual attitudes to the institutional conditions necessary for sustainable entrepreneurial outcomes. High-quality, context-sensitive pedagogy emerged as a clear program strength. To strengthen local development outcomes the study recommends: (a) decentralizing training delivery to reduce travel burdens, (b) establishing structured post-training incubation and market linkage mechanisms that leverage existing tribal cooperatives and local village councils (Lumbas) to create sustainable market chains for solar products, (c) adopting gender-responsive measures such as flexible scheduling, childcare provisions, and targeted support for women participants, and (d) integrating elements of traditional knowledge into training curricula to improve engagement among older community members. Future policy should recognize that individual skill development, while necessary is insufficient without parallel institutional investments in market access, supply chain development and community-based enterprise support structures. By addressing both individual competence and institutional conditions, renewable energy skill development initiatives can more effectively contribute to inclusive and sustainable local development in geographically marginal regions.

## Data Availability

The raw data supporting the conclusions of this article will be made available by the authors, without undue reservation.
