# Correction: Attitude of trainees toward tribal entrepreneurship training on solar lantern development in Ladakh: a descriptive study

**DOI:** 10.3389/fsoc.2026.2005255

**Published:** 2026-10-06

**Authors:** Mohd Rafee, Surendra Kumar Gotherwal, Tsewang Dolma, Anamika Sharma, Ruhana Rafiq, Stanzin Khenrab

**Affiliations:** 1 Department of Commerce and Management, University of Ladakh, Leh, India; 2 Ministry of Electronics and Information Technology (MeitY), New Delhi, India; 3 G.B. Pant University of Agriculture and Technology, Pantnagar, India; 4 Sher-e-Kashmir University of Agricultural Sciences and Technology of Jammu, Jammu, India; 5 Krishi Vigyan Kendra-Leh, University of Ladakh, Leh, India

**Keywords:** employment generation, Ladakh, renewable energy, skill development, tribal entrepreneurship, vocational training

The correct corresponding authors are Mohd Rafee and Tsewang Dolma.

The original version of this article has been updated.

